# Vestibular migraine

**DOI:** 10.1590/0004-282X-ANP-2022-S111

**Published:** 2022-08-12

**Authors:** Viviane Passarelli Ramin Silva, Luiz Henrique Martins Castro, Marcelo Calderaro

**Affiliations:** 1 Universidade de São Paulo, Faculdade de Medicina, Hospital das Clínicas, Ambulatório de Cefaleia, São Paulo SP, Brazil.; 2 Universidade de São Paulo, Faculdade de Medicina, Hospital das Clínicas, Departamento de Neurologia, São Paulo SP, Brazil.

**Keywords:** Headache, Migraine Disorders, Cefaleia, Transtornos de Enxaqueca

## Abstract

Vestibular migraine (VM) remains an underdiagnosed condition, often mistaken with brainstem aura. VM is defined by recurrent vestibular symptoms in at least 50% of migraine attacks. Diagnosis is established by clinical criteria based on the International Classification of Headache Disorders (ICHD-3). Estimated prevalence of VM is 1 to 2.7% of the adult population. Vestibular symptoms usually appear after the headache. VM pathophysiology remains poorly understood. Vertigo may occur before, during, after the migraine attack, or even independently, and may last seconds to hours or days. Pathophysiological mechanisms for VM are still poorly understood and are usually extrapolated from migraines. Differential diagnoses include Ménière's disease, benign paroxysmal positional vertigo, brainstem aura, transient ischemic attack, persistent perceptual postural vertigo, and episodic type 2 ataxia. Specific treatment recommendations for vestibular migraine are still scarce.

## RESUMO

Migrânea vestibular (MV) é pouco diagnosticada e comumente confundida com aura de tronco. A MV, definida por sintomas vestibulares recorrentes em até 50% das crises de migrânea. O diagnóstico baseia-se em critérios clínicos, descritos no *International Classification of Headache Disorders* (ICHD-3). Estima-se prevalência de MV em 1 a 2.7% da população adulta. Sintomas vestibulares geralmente ocorrem mais tardiamente comparados à cefaleia. A vertigem pode surgir antes, durante, depois, ou mesmo independentemente da crise de enxaqueca, com duração de segundos a horas ou dias. Pouco se conhece acerca da fisiopatologia da MV, que é geralmente extrapolada dos conhecimentos sobre migrânea. Diagnósticos diferenciais incluem: Doença de Ménière, Vertigem posicional paroxística benigna, aura de tronco, ataque isquêmico transitório, vertigem postural perceptual persistente e ataxia episódica tipo 2. Especialistas recomendam o mesmo tratamento que para enxaqueca. Estudos sobre o tratamento específico da migrânea vestibular ainda são escassos.

## INTRODUCTION

Vestibular migraine (VM) is defined by recurrent vestibular symptoms occurring in at least 50% of migraine attacks, lasting hours to days. Less than 10% vestibular migraine patients meet diagnostic criteria for brainstem aura. Vestibular symptoms can be more limiting than headache. VM remains an underdiagnosed condition. Knowledge about VM is largely extrapolated from migraine, and studies specifically addressing VM are scarce. 

## EPIDEMIOLOGY

Understanding the epidemiology of VM is limited by lack of biological markers, and by the fact that the diagnosis is established solely on clinical grounds. Using current criteria, vestibular migraine would be the most common cause of vertigo, with a prevalence between 1 to 2.7% of the adult population^1^. Age at onset of vestibular migraine (38) is usually later than migraine (23)^2^. Patients may not present headaches for many years, until the onset of vestibular symptoms^3^. VM is more prevalent in women (4:1)^1^ and approximately two thirds of patients will report a family history of migraine^2^.

## PATHOPHYSIOLOGY

**Figure 1.**
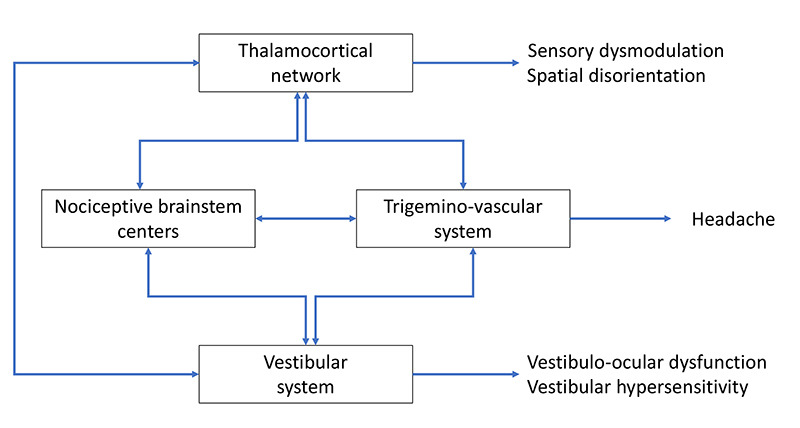
Vestibular migraine mechanisms (adapted from Huang TC - Vestibular migraine: An update on current understanding and future directions) 1.

Pathophysiology of vestibular migraine remains poorly understood (Figure 1). In addition to trigeminovascular dysfunction, considered the primary migraine mechanism, vestibular hyperexcitability, calcium voltage channel dysfunction, temporoparietal structural and functional changes also appear to play a role in VM. 

Possible mechanisms involved in the pathogenesis of vestibular migraine. Abnormal sensory modulation or integration within the thalamo-cortical network could result in dizziness and spatial disorientation. Hyperactivity within the trigeninovascular system and nociceptive brainstem centers could result in headache. Altered activity in the vestibular system could lead to transient vestibulo-ocular dysfunction or vestibular hypersensitivity associated with migraine features.

### Thalamocortical network disfunction

Thalamocortical network dysfunction is well established in migraine. (Goadsby, 2017). Thalamic nuclei activation modulates trigeminovascular input and other nociceptive information. Cortical hyperexcitability lowers migraine attack threshold in some patients^4^. VM patients are more sensitive to motion due to increased sensitivity to stimuli, and also display a lower threshold for perception of changes in body position, and more discomfort after oculocephalic reflex maneuvers, as well as more spatial perception errors. Ballet dancers, on the other hand, display higher threshold for vestibular output, and less cerebellar gray matter, suggesting that exercise interferes with vestibular processing^5^. 

### Vestibular hyperexcitability

 Painful trigeminal stimulation in migraine patients elicits new or worsening pre-existing peripheral nystagmus, suggesting vestibular hyperexcitability and predisposition to vertigo. Labyrinth dysfunction may be explained by ion calcium channel dysfunction, vasospasm, or central vestibular system dysfunction through brainstem nuclei activation^6^. 

### Voltage gated calcium channels (VGCC)

Clinical similarity between vestibular migraine and episodic type 2 ataxia, suggests shared pathophysiological mechanisms, such as voltage-gated calcium channel (CACNA1A) changes, also found in familial hemiplegic migraine^3^.

### Structural/functional temporoparietal changes

Temporoparietal structural and functional changes may also play a role in VM. FDG-PET studies during VM attacks show increased metabolism in these areas, underscoring the role of the vestibulo-thalamo-cortical pathway in VM^7^.

## DIAGNOSTIC CRITERIA

VM diagnosis is based on clinical criteria. 

Diagnostic criteria of VM^8^:

### Vestibular migraine [International Classification of Headache Disorders (ICHD-3) and International Classification of Vestibular Disorders (ICVD)]

- At least five episodes fulfilling criteria C and D;
- Current or past history of migraine with or without aura.
- Vestibular symptoms of moderate to severe intensity, lasting between five minutes and 72 hours;
- At least 50% of episodes are associated with at least one of the following three migrainous features:

1. *Headache with at least two of the following four characteristics:*

- Unilateral location;
- Throbbing quality;
- Moderate to severe intensity;
- Worsening with routine physical activity.

1. *Photophobia and phonophobia*
2. *Visual aura*

- Not better accounted for another ICHD-3 diagnosis or another vestibular disorder.

### Probable vestibular migraine (ICVD)

- At least five episodes of vestibular symptoms of moderate to severe intensity, lasting five minutes to 72 hours;
- Only one of the B and C criteria for vestibular migraine is fulfilled (migraine history *or* migraine features during the episode);
- Not better accounted for another vestibular or ICHD diagnosis, and presence of vestibular symptoms defined by Bárány Society’s Classification of Vestibular Symptoms, including^9^:

- spontaneous vertigo;
- internal vertigo, a false sensation of self-motion;
- external vertigo, a false sensation that visual surrounding is spinning or flowing.
- positional vertigo, occurring after head position changes;
- visually-induced vertigo, triggered by complex or large moving visual stimulus;
- head motion-induced vertigo, occurring during head motion;
- head motion-induced dizziness with nausea; dizziness is characterized by sensation of disturbed spatial orientation; other forms of dizziness are currently not included in the classification of vestibular migraine.

An isolated symptom is sufficient to characterize an episode. 

Vestibular symptoms are rated as “moderate”, interfering but do not hindering daily activities, and as “severe” when daily activities must be interrupted^9^.

The criteria underscore the importance of a directed clinical history of vertigo to accurately identify symptoms, leading to diagnosis.

## CLINICAL PRESENTATION

**Figure 2.**
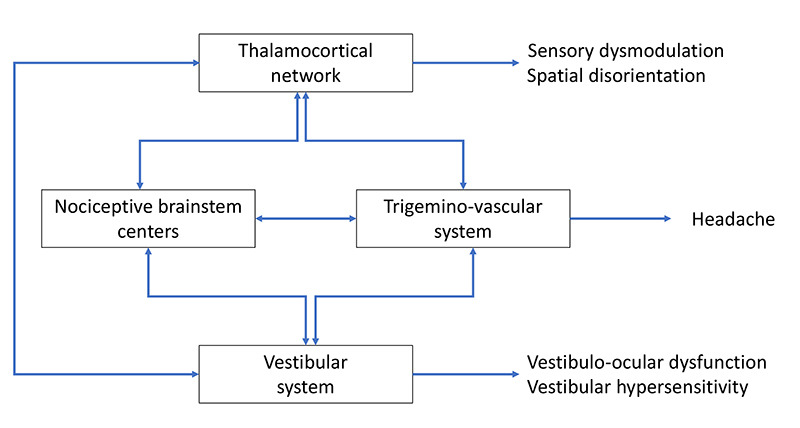
Vestibular symptoms during a migraine attack (adapted from Stolte B - Vestibular Migraine)^10^.

 Vertigo can antecede or may occur during, after, or even independently of the migraine attack, occurring in up to 30% of episodes, rendering diagnosis more challenging (Figure 2).

 Many patients consider vestibular symptoms as the most disabling feature of VM. Most patients report more than one vestibular symptom during attacks^1^, and up to 30% of VM episodes may be unaccompanied by headache.

 Episode duration follows the “30% rule”^10^.

- 30% last minutes
- 30% last hours
- 30% last days
- 10% remaining last seconds.

Auditory symptoms were reported in 40% of VM patients. the most common being sensation of blocked ear. Since symptoms are nonspecific, they were not included in the diagnostic criteria^3^. Presence of these symptoms may lead to misdiagnosis, especially of Ménière’s disease.

 Other VM symptoms include nausea, vomiting, prostration, imbalance and motion sickness^9^. Triggers do not differ from migraine: sleep deprivation, stress, menstrual cycle, food, weather changes and light.

 Psychiatric comorbidities are very common in VM patients (50% of cases), similar to migraine; most common symptoms are depression, anxiety and insomnia^1^.

 Several findings on physical examination, none specific, can confound diagnosis, such as presence of spontaneous or positional nystagmus consistent with central or peripheral nystagmus. Nystagmus can be evoked by positional maneuvers, and are slower and more persistent compared to BPPV nystagmus. Table 1 depicts the main neurological features, during and between VM attacks. Physical exam may be normal.

**Table 1.** Clinical findings in VM patients (Adapted from Sohn JH - Recent Advances in the Understanding of Vestibular Migraine)

Neurological findings
*Between attacks*
Gaze-induced nystagmus (27%) and spontaneous nystagmus (11%)
Persistent positional nystagmus and positional nystagmus (12-28%)
Vertical (48%) and/or horizontal (22%) saccadic pursuit
Subtle saccadic pursuit 20-63% on follow-up study (over 9 years)
Unilateral canal paresis (8-22%)
Bilateral vestibular failure (11%)
Low-frequency, mild cochlear loss (3-12%()
Mild bilateral sensorineural hearing loss (18%) on follow-up study (over 9 years)
*During Attacks*
Spontaneous nystagmus (19%) and nystagmus elicited by horizontal headshaking (35%)
Low-velocity, sustained, central positional nystagmus (100%)
Pathologic nystagmus with spontaneous or positional nystagmus (70%)
Central vestibular dysfunction (50%)
Peripheral vestibular dysfuntion (15%)
Unclear, mixture (35%)

## DIFFERENTIAL DIAGNOSES

 The main differential diagnosis is Ménière's disease. Symptom duration is similar, ranging from 20 minutes to 12 hours, accompanied by tinnitus and hearing loss. Low frequency hearing loss seen in more advanced stages of Ménière (inverted U peak in audiometry) may aid in diagnosis. Brain MRI can be useful to rule out other causes of vestibular symptoms, and to disclose endolymphatic hydrops, found in, 90% of Ménière's cases^12^. Migraine prevalence is twice more common in Ménière's disease patients^10^.

 Benign paroxysmal positional vertigo (BPPV) is another common cause of recurrent acute vertigo. Differentiation of BPPV and VM is difficult, and may only be possible by the finding of specific findings in neurological examination compatible with BPPV. Symptom duration may allow differentiating these conditions. In BPPV, symptoms last for weeks to months, and recur only after months or years. In VM, on the other hand, symptoms last hours to days, and recur several times a month or year^10^. Positional maneuvers are key to differentiate both conditions. Additionally, BPPV and migraine can coexist^13^. 

 Migraine with brainstem aura is commonly confused with VM, but has different diagnostic criteria. Although different, both can coexist. Migraine with aura must present two or more brainstem symptoms and/or signs (vertigo, hearing loss, dysarthria, tinnitus, diplopia, gait imbalance and decreased level of consciousness). Symptoms must be reversible, lasting from five to 60 minutes. Less than 10% of VM patients meet criteria for brainstem aura. Diagnostic criteria for brainstem aura according to ICHD-3 are the following:

1. Attacks fulfilling criteria for*Migraine with aura*and criterion below
2. Aura with both of the following:

- at least two of the following fully reversible brainstem symptoms:

- dysarthria^a^;
- vertigo^b^;
- tinnitus;
- hypacusis^c^;
- diplopia^d^;
- ataxia not attributable to sensory deficit;
- decreased level of consciousness (GCS ≤13)^e^.

- no motor^f^or retinal symptoms.

Notes:

^a^Dysarthria must be distinguished from aphasia.

^b^Vertigo does not include and should be distinguished from dizziness.

^c^This criterion is not fulfilled by sensation of a blocked ear.

^d^Diplopia does not include (or exclude) blurred vision.

^e^Glasgow Coma Scale (GCS) score assessed during admission; alternatively, deficits described by the patient allow GCS estimation.

^f^When motor symptoms are present, code as 1.2.3*Hemiplegic migraine*.

 Former phobic vertigo and current PPPV (persistent perceptual postural vertigo) should also be included in the differential diagnosis. Symptoms in these cases are nonspecific, such as empty headedness and malaise^14^. Episodes are recurrent, but tend to be more situational, leading to avoidance behavior. PPPV is commonly associated with psychiatric diagnoses, such as phobic anxiety disorder or depression and catastrophic thinking.

 Transient ischemic attack should be included in the differential diagnosis, especially if the initial episode is of sudden onset and if there is associated imbalance, and in older patients with cardiovascular comorbidities^9^.

 Type 2 episodic ataxia, although rare, should be included in the differential diagnosis. Type 2 episodic ataxia is a genetically inherited disease, associated CACNA1A channel changes, and is characterized by recurrent and disabling episodes of imbalance, vertigo and ataxia, that are induced by physical exertion or emotional stress. Episodes can be accompanied by headache in up to half of the cases. Outside attacks patients may display downbeat nystagmus. Head MRI may show mild anterior cerebellar vermis atrophy. Acetazolamide is the usual treatment^15^. Genetic testing confirms diagnosis. 

Table 2displays differential diagnoses for vestibular migraine.

**Table 2.** Differential diagnosis of vestibular migraine (adapted from Stolte B - Vestibular Migraine)

1. Ménière’s disease
2. Somatoform vertigo (primary or secondary that develops after vestibular vertigo
3. Benign paroxysmal positional vertigo (BPPV)
4. Posterior circulation transient ischemia (TIA)
5. Syncope or orthostatic hypotension
6. Vestibular paroxysmia
7. Episodic ataxia type 2

 Diagnosis of vestibular migraine is based on clinical findings. In specific situations where clinical overlap between diagnoses is suspected, additional tests may be requested.

Audiometry, electrocochleography and caloric testing are normal in most VM patients, when performed outside attacks^16^. In Ménière's disease , caloric testing, is usually abnormal and audiometry shows an inverted U peak in more advanced stages.

## TREATMENT

 Few studies report specific treatment recommendations for vestibular migraine. A search of clinical trials in February 2022 showed 18 clinical trials, six of which were stiil in the recruitment phase, five were completed and two were interrupted. From a practical standpoint, experts recommend the same treatment as for migraine. Antiemetics may be beneficial during attacks

 A randomized clinical trial showed a possible superiority of zolmitriptan^17^ in reducing vertigo intensity from severe/moderate to mild or absent two hours after medication intake. Design limitations and limited study power do not allow a definite conclusion, and further studies are needed.

 A rizatriptan trial for vestibular migraine was initiated in UCLA in late 2021. The trial is still underway and results are not yet available.^18^ A 2010 clinical trial using rizatriptan showed reduction in motion-induced sickness in patients with migraine, but this trial was not specifically designed for patients with vestibular migraine. A retrospective chart review suggested that nortriptyline and topiramate were effective in preventing VM^19^. Confirmatory clinical trials are lacking.

 Non-pharmacological treatment - vestibular rehabilitation - is usually recommended, and is probably effective, since there seems to be a protective effect of ballet training on modulation of the vestibular system in dancers, rendering them less sensitive to movement induced vertigo, as previously mentioned^5^.

 Although avoiding exposure triggers and caffeine cessation are often recommended, these measures display low efficacy on VM and may negatively impact on quality of life, and, therefore, should not be recommended.

Table 3shows a summary of the main abortive and preventive medications used in vestibular migraine, in addition to non-pharmacological treatment.

**Table 3.** Treatment options for Vestibular Migraine (adapted from Sohn JH. Recent Advances in the Understanding of Vestibular Migraine)^11^.

Acute medications		
Zolmitriptan	2.5mg oral	Randomized controlled trial
Rizatriptan	10mg oral	Randomized controlled trial, motion sickness
Prophylatic medications		
Propranolol	160mg, 40-60mg	Retrospective cohort analysis
Propranolol/venlafaxine	40-160mg/27.5-150mg	Prospective, randomized, controlled clinical trial
Metoprolol	150mg, 100-200mg	Retrospective cohort analysis
Amitryptiline	100mg, 10mg	Retrospective cohort analysis
Nortriptyline	27-75mg	Open-label, chart review
Valproic acid	600mg, 600mg	Retrospective cohort analysis, cohort study, vestibulo-ocular reflex
Topiramate	50mg, 50-100mg	Retrospective cohort analysis, open-label chart review
Lamotrigine	75mg	Retrospective cohort analysis
Flunarizine	5mg, 5-10mg, 5-10mg	Retrospective cohort analysis, retrospective
Cinarizine	37.5-75mg	Retrospective, open-label
Cinarizine + dimenhydrate	20mg and 40mg	Observational trial
Acetazolamide	500mg	Retrospective cohort study
Magnesium	400mg	Retrospective cohort analysis
Clonazepam	0.25-1mg	Retrospective cohort analysis
Non-medical treatments		
Vestibular rehabilitation	5 therapy sessions over nine weeks	Uncontrolled observational trial
Caffeine cessation	4 to 6 weeks	Retrospective, observational trial

 Vestibular migraine is a common and often underdiagnosed condition. Recognizing VM is key to adequately managing this disorder. Some VM episodes present with isolated vestibular symptoms, without headache. Careful history taking should disclose a history of associated migraine headaches..

 Therapeutic measures are currently based in usual migraine treatment options. Future studies should provide more information regarding management of VM symptoms. 
